# Extended, Double-Pedicled Facial Artery Musculomucosal (dpFAMM) Flap in Tongue Reconstruction in Edentulous Patients: Preliminary Report and Flap Design

**DOI:** 10.3390/medicina57080758

**Published:** 2021-07-26

**Authors:** Michał Gontarz, Jakub Bargiel, Krzysztof Gąsiorowski, Tomasz Marecik, Paweł Szczurowski, Jan Zapała, Grażyna Wyszyńska-Pawelec

**Affiliations:** Department of Cranio-Maxillofacial Surgery, Jagiellonian University Medical College, 30-688 Cracow, Poland; jakub.bargiel@uj.edu.pl (J.B.); krzysztof.gasiorowski@uj.edu.pl (K.G.); tomasz.marecik@uj.edu.pl (T.M.); pawel.szczurowski@uj.edu.pl (P.S.); jan.zapala@uj.edu.pl (J.Z.); grazyna.wyszynska-pawelec@uj.edu.pl (G.W.-P.)

**Keywords:** FAMM flap, facial artery musculomucosal flap, bozola flap, buccinator myomucosal flap, tongue reconstruction, tongue cancer, tongue squamous cell carcinoma

## Abstract

*Backgroundand Objectives*: The reconstruction of tongue defects after cancer resection is challenging for reconstructive surgeons. The facial artery musculomucosal (FAMM) flap and the myomucosal buccinator flap (Bozola flap) are important tools in the reconstruction of intraoral defects. In this study, we describe the combination of both flaps—the extended, double-pedicled FAMM (dpFAMM) flap—and present clinical results of the reconstruction of moderate tongue defects in edentulous patients. *Materials and Methods*: a tongue defect, after squamous cell carcinoma excision, was reconstructed with the dpFAMM flap in 5 patients. Most of them received postoperative radiotherapy. *Results*: the healing process was uneventful in all patients. We did not observe flap necrosis or venous congestion. Tongue mobility, speech and swallowing were satisfactory. *Conclusions*: In conclusion, the dpFAMM flap is a good alternative in the reconstruction of moderate defects of the lateral part of the tongue. The flap is easy to harvest and has a good vascularity. This is a predictable method of reconstruction, especially for elderly patients with numerous comorbidities.

## 1. Introduction

The tongue is a muscle fold covered with mucosa and is the most movable part of the oral cavity, responsible for many functions such as speech, swallowing and taste sensation. The reconstruction of the tongue has always been a challenge. The main goal of reconstructions has always been to preserve the patient’s speech and swallowing. In the literature, many techniques have been advocated for this purpose, from primary closure to local, regional and free flaps, depending on the defect size [1]. Most commonly, tongue defects are caused by the excision of squamous cell carcinoma (SCC). Tongue SCC is characterized by a high risk of nodal metastasis and poor overall survival [2]. According to Mannelli et al., surgical classification tongue defects are divided into five groups in ascending order of reconstructive complexity [3]. The smallest defects of the tongue border (type 1) can be primarily closed, moderate defects (type 2) without crossing the midline can be reconstructed with local or free flaps, and microsurgical reconstructions are the gold standard for the largest defects (type 3–5) [3]. Immediate tongue reconstruction should be performed after primary tumor resection, and its size affects the severity of function impairment.

Defects of the oral cavity might be reconstructed by local flaps, such as the facial artery musculomucosal (FAMM) flap, firstly described by Pribaz et al. in 1992 [4]. Traditionally, the FAMM flap is an axial flap based on the facial artery, and 2.5 to 3 cm of width, is used in the two-stage method of reconstruction. The facial vein is rarely harvested with the flap. Venous drainage is possible by a submucosal plexus in the pedicle, which is a width of at least 2 cm, located in the area of the second and third lower molar teeth [4,5]. According to Ayad and Xie’s study, the FAMM flap is usually used in the reconstruction of defects post-tumor ablation, followed by for the cleft palate and osteoradionecrosis [5]. Partial necrosis of FAMM flaps is observed in 12.2% and total necrosis in 2.9% of cases [5]. Another local flap is the Bozola flap, based on buccal vessels, which is mainly used in the reconstruction of defects of the upper part of the oral cavity, e.g., the palate [6]. The Bozola flap also can be used in tongue reconstruction, with good clinical results recorded [7,8]. Local flaps used for the reconstruction of the oral cavity provide an alternative to the free flap reconstruction of postoperative moderate tongue defects. The most important advantages are the shorter time of the procedure, which can be done by one team of surgeons, and also both the shorter time and lower costs of hospitalization [9]. Another strength of using local flaps is the psychological aspect for patients, who are saved from additional external scars and the more severe donor site morbidity of microsurgical reconstructions. The use of local flaps circumvent the problems of excessive bulk and hair growth seen with the use of regional and free flaps [10]. The major limitation of using local flaps in tongue reconstructions is the relatively small size of the flaps, which cannot reach and reconstruct precisely the apex of the tongue.

The original FAMM flap is usually used to reconstruct the defects of the floor of the mouth, palate, alveolar ridge, lip and oropharynx [5]. The narrow FAMM flap comprises insufficient material to reconstruct type 2 tongue defects. Our modification of the FAMM flap by preserving the venous drainage through the facial vein and also the buccal vessels, similarly to the Bozola flap, allows for flap extension and enables the reconstruction of almost half of the tongue in the lateral region. The flap harvest is simple, with minimal donor site morbidity, such as transient facial nerve paresis or moderate cheek deformity. The flap has the perfect color and structure, matching the reconstructed tongue.

In this study, we describe a step by step procedure for harvesting the extended, double-pedicled FAMM (dpFAMM) flap, and present a preliminary report of clinical results in the reconstruction of moderate tongue defects (type 2 according Mannelli et al.’s classification) in edentulous patients. Additionally, the strengths and limitations of the flap are discussed.

## 2. Materials and Methods

Between January 2021 and June 2021, 5 patients with SCC of the tongue had their tongues reconstructed with dpFAMM flaps in the Department of Cranio-Maxillofacial Surgery of the Jagiellonian University in Cracow. All of them were edentulous with multiple comorbidities and usually required postoperative radiotherapy. The patients’ characteristics are presented in Table 1.

### 2.1. Flap Anatomy

**Figure 1:**
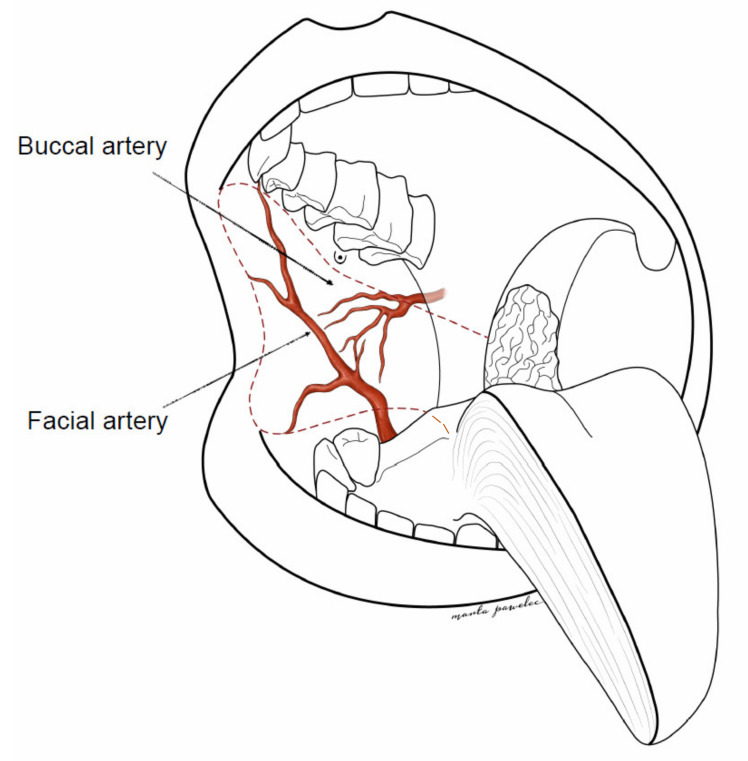
Extended, double-pedicled facial artery musculomucosal (dpFAMM) flap design—red dotted line.

Anatomically, the buccal region consists of six layers. The outer layer is skin; the subcutaneous layer is adipose tissue; the buccopharyngeal fascia, the buccinator muscle, and the submucosal tissue are minor salivary glands; the most inner layer is the buccal mucosa. The buccal region vascularization is derived from the branches of the facial artery that emerge at the level of the anterior edge of the masseter muscle (inferior second, third molar), and run along the outer surface of the buccinator muscle obliquely upward towards the nasal ala, about 1.5 to 2 cm laterally from the oral commissure. The facial vein is located approximately 1.5 cm distal to the artery and runs more parallel to the anterior edge of the masseter muscle towards the parotid papilla [5]. The second source of vascularization of the buccal region comes from the buccal artery, which is a branch of the maxillary artery (Figure 1) [7].

The buccal artery reaches the posterior half of the muscle, goes under the pterygoid muscle, and runs forward and downward to the buccinator muscle. The venous drainage is based on several veins, some are comitantes to arteries, others are not. All of them are tributaries to either an anterior collector (the facial vein) or a posterior collector (the pterygoid plexus and the maxillary vein) [7].

The dpFAMM flap consists of the buccal mucosa, the submucosal tissue, the buccinator muscle and the facial and buccal vessels. The facial vessels comprise the lateral pedicle of the flap, and the buccal vessels with mucosa in the area of the pteryrigomandibular raphe comprise the medial pedicle. The anterior border of the flap is 1 cm distal to the oral commissure, the upper limitation is 0.5 cm below the parotid papilla, the length of the flap is 6–7 cm, the width is 4–5 cm in the anterior region, and the width of the mucosal pedicle in the area of the pteryrigomandibular raphe is 3 cm (Figure 1). There is also a possibility to widen the central part of the flap by cutting the distal part of Stensen’s duct and its transposition, similarly to the Bozola flap technique [8].

### 2.2. Surgical Technique

**Figure 2:**
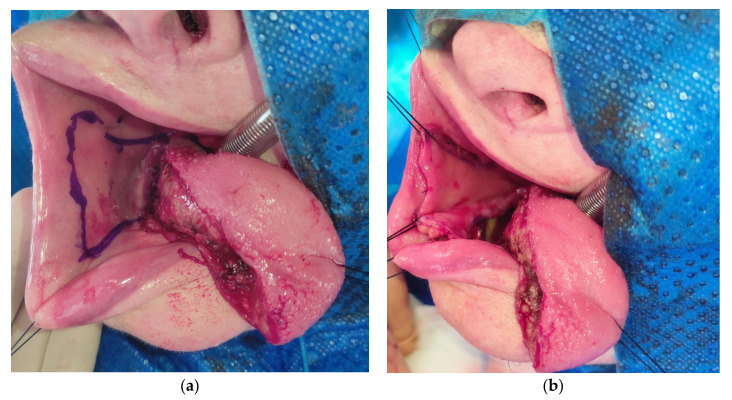
(**a**) The dpFAMM flap marked on the healthy buccal mucosa; (**b**) the incision of mucosal, submucosal tissue and the buccinator muscle in the dpFAMM flap.

All patients signed the informed consent prior to the treatment. Selective bilateral neck dissection (levels I-IV ipsilateral and I-III contralateral) was performed in patients via a horizontal neck fold incision. During neck dissection, facial vessels with the marginal branch of the facial nerve were preserved. An intaoral excision of the tongue squamous cell carcinoma with adequate margin was performed. In four cases also, the distal part of the floor of the mouth was excised. The range of the flap was marked on the healthy buccal mucosa (Figure 2).

**Figure 3:**
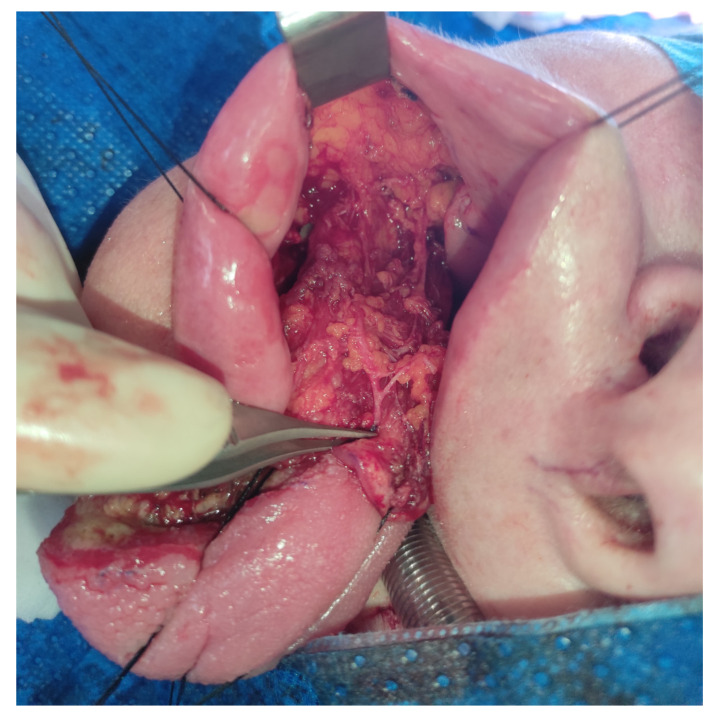
Harvesting of the dpFAMM flap. Visible are the buccinator muscle with the facial artery (pointed by tweezers) and the bulking buccal fat pad.

The anterior border of the mucosal pedicle should be located at the level of the second lower molar, and the incision over the alveolar crest should reach the tongue’s defect. It is contraindicated to widen the mucosal pedicle forward into the area of the premolars and the canine due to the difficulty of donor side closure. In this area, the flap constitutes only the mucosa of the lower gingiva and the periosteum of the mandible without the buccinator muscle. Those tissues are unsuitable for the reconstruction of the lateral border of the tongue. Before flap harvesting, it is advisable to infiltrate the soft tissues with saline. The facial artery is easily found by palpation or could be Dopplered. The incision starts through the mucosa, the submucosal tissue and the buccinator muscle in the anterosuperior part of the flap. After finding the superior labial artery, it is ligated and cut. Then, following the superior labial artery, the facial artery is found, and is also ligated and cut in the upper part of the flap. It is advisable to suture the stump of the facial artery to the buccinator muscle and the mucosal edge of the flap to reduce the risk of the artery being torn from the flap muscle. At this stage, a deep dissection takes place at the border of the buccinator muscle and along the facial artery. The dissection border is the adipose tissue, where care should be taken to preserve the branches of the facial nerve. In the upper part of the flap, near the parotid papilla, the facial vein is reached, which is also ligated, cut and sewn into the upper edge of the flap. In this area also, the buccal fat pad and the buccal vessels are visible and saved (Figure 3).

**Figure 4:**
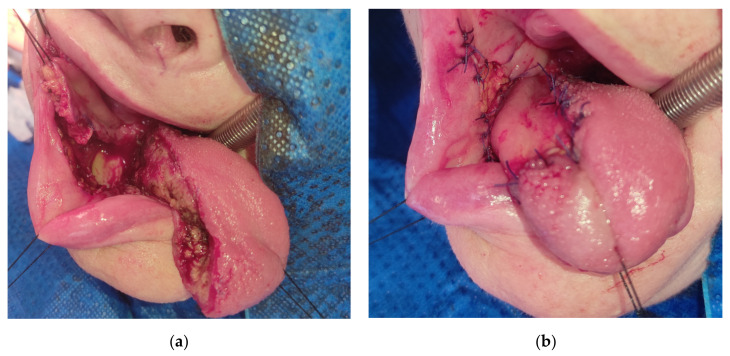
(**a**) The harvested dpFAMM flap with visible mandible exposure; (**b**) tongue reconstruction with the dpFAMM flap. Donor site closed by direct sutures in the anterior part and by the buccal fat pad in the posterior part.

Then, a subperiosteal dissection below the flap’s pedicle is performed over the mandibular body and ramus on the outer and inner sides. For better mobilization of the flap, it is advisable to cut the periosteum, which can be performed by an extraoral approach to limit the risk of the facial vessels damage. The harvested flap is pediculated on the facial vessels in the disto-lateral part and on the buccal vessels with mucosa in the area of the pteryrigomandibular raphe in the disto-medial part. The flap is then rotated 180° towards the tongue’s defect. The distal part of the flap’s mucosal pedicle can be used to reconstruct the tongue base, while the anterior part of the flap reaches the apex of the reconstructed tongue (Figure 4).

**Figure 5:**
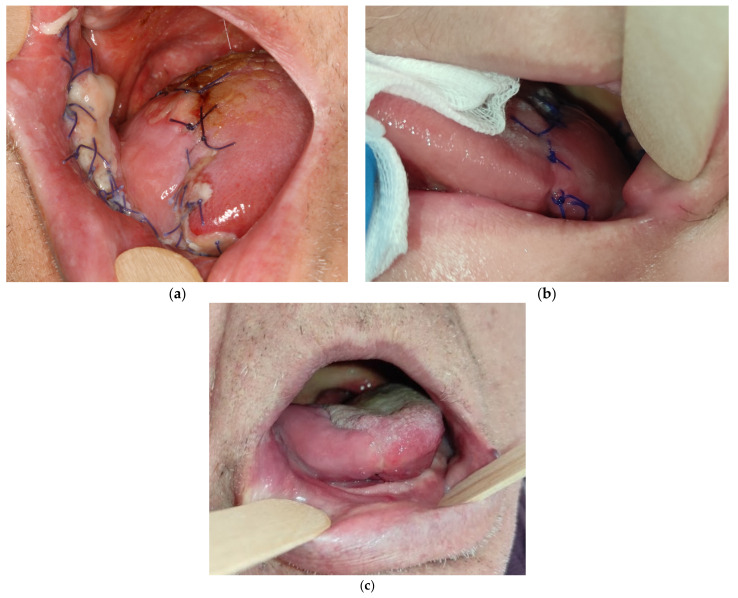
Results: (**a**) 79-year-old patient 5 days after the reconstruction; (**b**) 49-year-old patient 10 days after the reconstruction; (**c**) final result 3 weeks after surgery in 74-year-old patient.

The anterior upper and lower part of the donor site is closed with direct closure, while the distal part is covered with the buccal fat pad. After surgery, prolonged intubation was carried on for one day. The tracheotomy was avoided in all cases. In the postoperative period, the patients were fed by a nasogastric tube for 10 to 14 days during the epithelialization of the buccal fat pad (Figure 5).

## 3. Results

The healing process was uneventful in all patients. We did not observe flap necrosis or venous congestion. Tongue mobility, speech and swallowing were satisfactory. However, all patients required exercises ordered by a speech therapist. Restriction in protrusion of the tongue with a deviation towards the operated side was observed in all patients. Proper mouth closure was achieved in every case. The oncological follow-up was performed according to National Comprehensive Cancer Network (NCCN) guidelines [11]. At present, we do not have long-term follow-up data of the patients after radiotherapy. Postradiotherapy fibrosis affecting the function of the tongue and the buccal area should be taken into consideration.

## 4. Discussion

During the COVID-19 pandemic, we have observed a greater number of patients admitted to hospitals in the advanced stages of cancer. Due to the often more limited access to operating theaters and intensive care units because of the pandemic, it is necessary to use alternative reconstructive methods to reduce the time of surgery and hospitalization. The use of the dpFAMM flap is an alternative to the microsurgical reconstruction of postoperative moderate tongue defects (up to post-hemiglossectomy defects), without the need for tracheotomy, especially in edentulous patients with multiple comorbidities.

**Figure 6:**
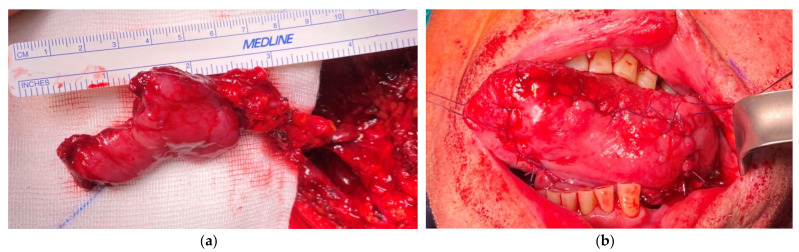
(**a**) The island FAMM flap with a facial artery and vein pedicle; (**b**) tongue reconstruction with the island FAMM flap.

The FAMM flap is an axial flap based on the facial artery and can be both superiorly or inferiorly based [4]. This flap is usually used to reconstruct small- and medium-sized defects of the oral cavity [5]. The traditionally inferiorly based FAMM flap has a mucosal pedicle with a width of 2 cm, comprises a two-stage procedure, and requires pedicle modeling 3 to 4 weeks after the first stage of reconstruction [5]. There are a number of technical modifications of this flap. Duranceau and Ayad presented the FAMM flap modification as a single-stage reconstruction of the floor of the mouth with the mucosal pedicle reaching the defect over the alveolar crest. In this modification, patients did not have lower molars and the width of the flap did not exceed 3 cm [12]. Another modification described by Zhao et al. is an island flap with a facial artery and a vein pedicle. This is also a one-step procedure which can be used for tongue reconstruction [13]. The island FAMM flap is tunnelled lingually under the mandibular body and can be used especially in patients with full dentition [14,15] (Figure 6).

However, the duration of island flap harvesting is longer and more difficult, and there is a higher risk of flap necrosis, mainly due to kinking of the pedicle. Moreover, in the case of both the FAMM and island FAMM flap, venous congestion is a quite often phenomenon in the postoperative period [5]. Also in the case of postoperative complications, such as neck hematomas, the risk of island flap necrosis is higher due to the clamping of the facial vein and its clotting, especially in patients chronically treated with dual antiplatelet therapy. The additional venous drainage through buccal veins and submucosal plexuses in the dpFAMM flap causes this flap to be more resistant to venous congestion and necrosis, even in patients with neck hematomas in the postoperative period. The island FAMM flap also is smaller in size and has a greater risk of injury to the marginal branch of the facial nerve during facial vessel pedicle dissection than the dpFAMM flap. The FAMM flap can also be used as a free flap in microsurgical reconstructions [16].

Another local flap used in small and medium sized defects of the oral cavity reconstruction is the Bozola flap, described in 1989, which is vascularized by the buccal vessels [6]. This flap can also be used to reconstruct the base and posterior part of the mobile tongue [7,8]. However, its limitations are its inability to expand the flap anteriorly upward and downward due to facial vessel vascularization in this area, and the risk of partial flap necrosis. The Bozola flap, similarly to the dpFAMM flap, can be widened in the central part by cutting the end of Stensen’s duct and its transposition [8].

The dpFAMM flap combines the advantages of both the FAMM and the Bozola flap, which allows its extension to be increased with sufficient venous drainage. Due to its extension in the anterior part, the dpFAMM flap can also be used for the tongue and floor of the mouth reconstruction. The flap has a perfect color and structure, matching the surrounding tissues. The procedure is shorter than microsurgical reconstructions and can be performed by one surgical team. Further, the patient does not require postoperative tracheotomy, and the hospitalization time is shorter. In addition, the patients do not have additional extraoral scars and the donor site is closed by direct closure with buccal fat pad transposition. A short postoperative hospitalization with a good healing process provides the possibility of early adjuvant radiotherapy. Other advantages of the dpFAMM flap include its ability to be used in the reconstruction of other areas of the oral cavity, such as the palate or the floor of the mouth. The use of a bilateral dpFAMM flap allows for the reconstruction of the floor of the mouth in the anterior region with the ventral surface of the tongue. In addition, the facial vessel preservation during the neck dissection guarantees better drainage and decreases of lymphedema in the face and neck area. Its main disadvantages include the inability to use this flap in the case of infiltration of the facial vessels by cancer metastasis. In these cases, the Bozola flap can be used. Another disadvantage is the need to remove the second and third lower molars to protect the vascular pedicle from occlusal interference or the use of bite blocks in patients with full dentition [8].

## 5. Conclusions

In conclusion, the dpFAMM flap is a good alternative for the reconstruction of moderate defects of the lateral part of the tongue (type 2). The flap is easy to harvest and has a good vascularity; therefore, the healing process is uneventful and, hospitalization time is short. This is a predictable method of reconstruction, especially for elderly patients with numerous comorbidities, who are often chronically treated with dual antiplatelet therapy. Due to its wide arch of rotation, the dpFAMM flap might be used in the reconstruction of other parts of the oral cavity, such as the floor of the mouth, the soft and hard palates, the alveolar ridge, or the oropharynx.

## Figures and Tables

**Table 1:** Characteristics of patients with squamous cell carcinoma of the tongue.

No	Age	Sex	cTNM	Neck Dissection	pTN	Grade	Comorbidities	Additional Treatment
1	74	M	T2N1M0	Bilateral SND	T3N1	G2	External iliac artery angioplasty with stent implantation, hypertension, atherosclerosis, cataract	PORT
2	64	F	T2N1M0	Bilateral SND	T3N0	G3	Stroke with left hemiplegia, hypertension, alcohol dependence syndrome, mitral valve regurgitation, stomach ulcer, cholecystolithiasis	PORT
3	79	M	T3N2cM0	Bilateral SND	T3N2b	G2	Senile dementia, diabetes mellitus, hypertension	PORT
4	83	F	T3N0M0 (recurrence after definitive radiotherapy)	No (patient after bilateral SND due to tongue SCC on the oposite side in 2019)	T3Nx	G2	After tongue SCC T3N1M0 resection with bilateral SND, radioterapy due to new foci of tongue SCC, chemotherapy due to breast cancer, hypertension	No
5	49	F	T3N1M0	Bilateral SND	T3N0	G1	No	PORT

M—male; F—female; SND—selective neck dissection; PORT—postoperative radiotherapy; cTNM: clinical Tumor, Node, Metastasis; pTN: pathological Tumor, Node; SCC: squamous cell carcinoma.

## Data Availability

Restrictions apply to the availability of these data. Data were obtained from patients treated at the Department of Cranio-Maxillofacial Surgery, Cracow, Poland, and cannot be shared, in accordance with the General Data Protection Regulation (EU) 2016/679.
